# Comparison of Risk Profiles, Nutrient Intake, and Kidney Function of Calcium Oxalate Stone Formers with and without Enteric Hyperoxaluria. A Matched Case-Control Study

**DOI:** 10.3390/nu18111788

**Published:** 2026-06-01

**Authors:** Charlotte Ernsten, Nikolai Spuck, Albrecht Hesse, Roswitha Siener

**Affiliations:** 1 University Stone Center, Clinic and Polyclinic for Urology and Pediatric Urology, University Hospital Bonn, University of Bonn, 53127 Bonn, Germany; s4cherns@uni-bonn.de (C.E.); albrecht-hesse@web.de (A.H.); 2 Institute for Medical Biometry, Informatics, and Epidemiology (IMBIE), University Hospital Bonn, University of Bonn, 53113 Bonn, Germany; nikolai.spuck@zi-mannheim.de; 3 Core Facility Biostatistics, Central Institute of Mental Health, Medical Faculty Mannheim, Heidelberg University, 68159 Mannheim, Germany

**Keywords:** kidney stones, urolithiasis, diet, calcium, oxalate, enteric hyperoxaluria, intestinal oxalate absorption, urine pH, kidney function, glomerular filtration rate

## Abstract

**Objectives**: This study compared the risk profiles, nutrient intake, and kidney function of calcium oxalate stone formers with and without enteric hyperoxaluria. **Methods**: Thirty-seven patients with calcium oxalate stone disease and enteric hyperoxaluria (cases) and 37 sex- and age-matched idiopathic calcium oxalate stone formers (controls) were enrolled. Patients did not receive any nutritional counseling prior to the start of the study, and they discontinued medications affecting urinary parameters four weeks before their study participation. Anthropometric, clinical, and metabolic parameters were recorded. Dietary and 24-h urinary variables were measured under the habitual diet and under a balanced, standardized diet. The [^13^C_2_] oxalate absorption and calcium loading tests were carried out. **Results**: The median [^13^C_2_] oxalate absorption was significantly higher in cases (14.8%) than in controls (8.9%). Under the balanced diet, hypocitraturia, hypomagnesuria, low urine volume and pH value were significantly more common in the case group, whereas hypercalciuria occurred more frequently in the control group, affecting 45.9% of controls and 5.4% of cases. Furthermore, the control group exhibited a greater reduction in urinary calcium excretion under the balanced diet. Urinary oxalate excretion and the ion-activity product index of calcium oxalate were significantly higher under both diets, with a greater decline observed in the case group under the balanced diet. The estimated glomerular filtration rate (eGFR) was lower in cases. A multivariable linear regression analysis revealed a significant association between urine pH and eGFR. **Conclusions**: Calcium oxalate stone formers with and without enteric hyperoxaluria benefit from a balanced diet and sufficient fluid intake. The reduction in urinary oxalate excretion and the biochemical risk of recurrent calcium oxalate stone formation were even more pronounced in patients with enteric hyperoxaluria. Particular attention should be paid to low urine pH, as it is hypothesized to be a potential indicator of impaired kidney function.

## 1. Introduction

Urolithiasis is a common urological disease worldwide that predominantly affects patients of working age and imposes a substantial burden on healthcare systems [1,2]. Calcium oxalate is the most common type of stone, with approximately 70% of calculi containing calcium oxalate as the main component [3,4]. Hyperoxaluria is considered a major predisposing factor for calcium oxalate stone formation [5]. Enteric hyperoxaluria is a metabolic disturbance occurring in malabsorptive bowel conditions associated with fat malabsorption, such as Crohn’s disease, exocrine pancreatic insufficiency, short bowel syndrome, and bypass surgery [6,7,8]. These conditions result in elevated urinary oxalate excretion, which is attributed to enhanced intestinal absorption of oxalate [9]. Due to excess loss of bile acids in the stool caused by impaired reabsorption in the ileum, absorption of fatty acids in the small intestine is diminished [6,10]. Consequently, calcium binds to unabsorbed fatty acids, resulting in a lower luminal calcium concentration [11]. As a result, larger amounts of free oxalate are available for absorption in the colon, the key intestinal segment of oxalate absorption [9,10,11,12]. Additionally, unabsorbed bile salts and fatty acids increase colonic permeability to oxalate, thereby facilitating oxalate absorption and increasing urinary oxalate excretion [12,13].

Patients suffering from urolithiasis are at increased risk of chronic kidney disease (CKD), which can ultimately lead to end-stage renal disease [14,15]. Kidney damage may occur as a consequence of stone formation, such as obstruction, of urological interventions for stone removal, and of underlying diseases that favor kidney stone formation [16,17]. Kidney stone formers with intestinal diseases are known to frequently manifest CKD [17,18]. A study of patients with Crohn’s disease demonstrated a 10-fold higher risk of developing CKD for patients with a history of urolithiasis than for those without kidney stones [19]. In the advanced stages of CKD, metabolic acidosis may occur, as the kidney loses the ability to maintain acid-base balance [20]. Metabolic acidosis has been observed to not only induce bone demineralization, muscle catabolism, insulin resistance, and hypertension, but also to promote the progression of CKD and to increase mortality [20,21]. It was hypothesized that hyperoxaluria may contribute to the development of oxalate nephropathy, which is defined as crystalline calcium oxalate deposition in renal tissue, causing tubular injury, inflammation, and fibrosis, thereby impairing kidney function [22,23,24].

Although enteric hyperoxaluria is known as a major risk factor for calcium oxalate stone formation, studies investigating the anthropometric, clinical, and metabolic characteristics, urinary risk profile, and dietary composition of calcium oxalate stone formers with enteric hyperoxaluria in comparison to idiopathic calcium oxalate stone formers are lacking, especially under controlled, standardized conditions. Moreover, there is a need for research into the risk of CKD in calcium oxalate stone formers, as well as the contributing factors. Therefore, the present study aimed to investigate the pathophysiologic differences related to intestinal absorption, acid-base status, and kidney function under a balanced, standardized diet, and to explore the impact of the habitual diet of the patients. Furthermore, the objective of this matched case-control study was to evaluate the factors associated with impaired kidney function in calcium oxalate stone formers with enteric hyperoxaluria and idiopathic calcium oxalate stone formers.

## 2. Materials and Methods

### 2.1. Study Participants

This matched case-control study enrolled adult women and men with a history of calcium oxalate stone disease. The inclusion criterion for both cases and controls was a documented calculus from a recent stone event containing at least 50% calcium oxalate. Stone composition was analyzed using Fourier transform infrared spectroscopy (FTIR) (PerkinElmer, Waltham, MA, USA).

Calcium oxalate stone formers with enteric hyperoxaluria and idiopathic calcium oxalate stone formers were recruited from the University Stone Center of the Department of Urology and Pediatric Urology at the University Hospital Bonn. The case group consisted of 37 calcium oxalate stone formers with a documented medical history of hyperoxaluria secondary to a previously diagnosed enteric disorder associated with malabsorption. The underlying diseases were: Crohn’s disease with bowel resection in 26 patients, chronic pancreatitis in two patients, and bariatric bypass surgery in one patient. In eight calcium oxalate stone formers in the case group, enteric hyperoxaluria was attributed to small bowel resection for other reasons, which included mesenteric vein thrombosis (two patients), ileus (two patients), inflammatory cecal tumor, ileal neuroendocrine tumor, benign small intestine tumor, and volvulus (one patient each) [7]. Hyperoxaluria, defined as urinary oxalate excretion of at least 0.450 mmol/24 h [25], had to have been diagnosed prior to the study under the patients’ habitual diet. The control group comprised 37 sex- and age-matched patients with idiopathic calcium oxalate stone disease. Exclusion criteria for the idiopathic calcium oxalate stone formers were conditions associated with enteric hyperoxaluria, primary hyperoxaluria, primary hyperparathyroidism, renal tubular acidosis, medullary sponge kidney, sarcoidosis, bone disease, vitamin D excess, and nephrocalcinosis [16].

All patients were admitted to the University Stone Center of the Department of Urology and Pediatric Urology at the University Hospital Bonn for an inpatient metabolic assessment under controlled, standardized conditions. The study participants were instructed to discontinue medications or supplements that could affect urinary risk factors for stone formation, such as calcium, magnesium, vitamin D, alkaline citrate, sodium bicarbonate, thiazides, allopurinol, or pyridoxine, at least 4 weeks prior to and during the study. Before enrollment, patients received no nutritional advice and were asked to adhere to their customary dietary habits. The study was approved by the Ethics Committee of the Medical Faculty of the University of Bonn (approval number 430/19), and informed consent was obtained from all participants.

### 2.2. Study Procedure

The following were obtained from the patients under their habitual diet: anthropometric and clinical characteristics, 24-h urinary parameters, and medical history. After precise instructions from trained staff, each study participant documented detailed information on the types and amounts of all foods and beverages in a 7-day dietary protocol while following their habitual diet. Computer program PRODI 5.3 (Nutri-Science GmbH, Freiburg, Germany) was used to calculate dietary composition. The oxalate content of all foods and beverages analyzed in the laboratory of the University Stone Center was entered into the database [26,27]. Dietary sodium intake was derived from 24-h urinary sodium excretion [28].

In the subsequent phase, stone formers were given a standardized, balanced diet comprising 2.5 L of beverages per day for 11 days [28,29]. Patients were advised to consume their allotted meals, and urine volume was measured to monitor fluid intake. Trained personnel supervised patient compliance. Study participants collected 24-h urine samples on their habitual diet and after 7 days on the balanced, standardized diet, when a metabolic steady state and constant urinary values were achieved [29]. Analysis of 24-h urine parameters was performed as previously described [30], and external laboratory quality certificates were available for urinary stone analysis and for each urine parameter. Reference ranges for metabolic abnormalities were adapted from the current guidelines of the German Society of Urology [31], except for urinary oxalate excretion, for which the threshold value refers to [25]. The software EQUIL2 was used to calculate the relative supersaturation of uric acid and calcium oxalate [32]. The Tiselius ion-activity product indices of calcium oxalate and uric acid were determined [29,33]. The estimated glomerular filtration rate (eGFR) was assessed according to the CKD-EPI equation for adults [34]. A blood gas analysis was performed using a Radiometer ABL 510 blood gas analyzer (Radiometer, Copenhagen, Denmark).

The calcium loading test was conducted on days 2 and 3, and the [^13^C_2_] oxalate absorption test on days 9 and 10 under controlled, standardized conditions [29]. The oral calcium loading test was carried out to diagnose the different forms of hypercalciuria [29]. Patients with urinary calcium excretion of at least 5 mmol/24 h on a balanced, standardized diet were evaluated. The [^13^C_2_] oxalate absorption test was conducted to investigate intestinal oxalate absorption of patients with enteric hyperoxaluria and idiopathic calcium oxalate stone formers [29,35]. Hyperabsorption of oxalate is defined as intestinal absorption greater than or equal to 10% [29,36].

### 2.3. Statistical Analysis

Case-control matching was performed using the case-control matching function of IBM SPSS version 29.0 (SPSS Inc., Chicago, IL, USA) with the matching variables sex and age, including a tolerance of 9 years for age. The non-parametric Wilcoxon signed rank test was used to assess differences in the metric variables between cases and matched controls and between the habitual diet and the balanced, standardized diet within cases and controls, respectively. Associations between the binary categorical variables and group (case or control) were investigated using the exact version of McNemar’s test. Summary statistics are expressed as medians with the corresponding interquartile ranges for the metric variables as well as absolute and relative frequencies for the categorical variables. Spearman’s rank correlation was used to calculate associations between variables. The occurrence of urinary abnormalities in cases and controls was compared using odds ratios. Multivariable linear regression analysis was performed based on the data of all stone formers in both groups to investigate the relationship between the total number of stone events, pH value, as well as oxalate excretion in 24-h urine (in mmol/24 h) under the balanced, standardized diet and eGFR. The model for eGFR additionally included the body mass index (BMI, in kg/m^2^), patient group (cases or controls), presence of hypertension (yes or no), and presence of diabetes mellitus (yes or no) as potential confounders. In order to apply regression analysis to the complete data set, 26 missing values for the total number of stone events and one missing value for BMI were imputed using predictive mean matching as implemented in the R package mice [37]. The regression model was fitted to 50 imputed data sets, and the results were pooled together. Standard errors for confidence intervals (CI) were calculated based on Rubin’s rules. All statistical tests were two-sided with a significance level of α = 0.05, not accounting for the effects of multiple testing. The analyses were intended to be explorative and were performed using SPSS version 29.0 and R version 4.5.1.

## 3. Results

### 3.1. Patient Characteristics

A total of 37 cases and 37 sex- and age-matched controls were enrolled in this study. Patient characteristics of both groups are depicted in Table 1. The case group comprised calcium oxalate stone formers with enteric hyperoxaluria, including 27 men (73%) and 10 women (27%). The underlying enteric conditions resulting in enteric hyperoxaluria were Crohn’s disease with bowel resection (70.3%), small bowel resection for other reasons (21.6%), such as ileus and mesenteric vein thrombosis, chronic pancreatitis (5.4%), and bypass surgery (2.7%) [7]. The control group consisted of 37 sex- and age-matched idiopathic calcium oxalate stone formers. The median age of the patients was 48 years in cases and 46 years in controls, ranging from 25 to 72 years in both groups. While the BMI was significantly higher in controls, no significant differences were detected regarding the frequency of cardiovascular diseases, type 2 diabetes, and gallstone disease/cholecystectomy.

Median [^13^C_2_] oxalate absorption was 14.8% in cases, which was significantly higher than the median value of 8.9% in controls. In addition, hyperabsorption of oxalate, defined as intestinal oxalate absorption of 10.0% or higher, was significantly more prevalent in the case group, affecting 77.4% of cases and 44.8% of controls. In contrast, patients in the control group were significantly more often diagnosed with hypercalciuria, defined as urinary calcium excretion of at least 5 mmol/24 h under the balanced, standardized diet, with absorptive hypercalciuria being the most common type. The median eGFR was significantly higher in the control group, with a median eGFR of 75.7 mL/min/1.73 m^2^ in cases and 90.7 mL/min/1.73 m^2^ in controls. While an eGFR below 60.0 mL/min/1.73 m^2^, corresponding to chronic kidney disease (CKD) ≥ stage 3, occurred in 29.7% of all cases, no stone formers in the control group presented with an eGFR below 60.0 mL/min/1.73 m^2^. Blood gas analysis revealed significantly lower median pH value, bicarbonate concentration, and base excess in the case group. Metabolic acidosis, defined as a bicarbonate concentration < 22 mmol/L, was noted in 16.7% of patients in the case group, without affecting any patients in the control group.

The medical history of urinary stone disease of the patients is shown in Table 2. The frequency of anatomical anomalies was similar in cases and controls, with kidney cysts being the most common abnormality. All patients except four in each group had active intervention for stone removal, with extracorporeal shock wave lithotripsy and ureteroscopy being the most frequent procedures.

### 3.2. Urine Composition

Urinary parameters of the cases and the matched controls under the habitual diet and the balanced, standardized diet are presented in Table 3. Under both diets, urine pH and the excretion of calcium, magnesium, sulfate, uric acid, and citrate were significantly lower in cases, while controls exhibited a significantly lower urinary excretion of ammonium and oxalate. The relative supersaturation of calcium oxalate, the ion-activity product index of calcium oxalate, as well as the ion-activity product of uric acid, were significantly higher in stone formers with enteric hyperoxaluria under the habitual diet and the balanced, standardized diet. Only under the free-choice diet was urinary potassium excretion lower in cases. The urinary chloride excretion and relative supersaturation of uric acid in cases exceeded those in the control group only under the balanced diet. No other significant differences were detected between cases and controls under both diets. Differences in changes in urinary parameters were detected for urinary calcium excretion, for which controls showed a greater median decline of 1.24 mmol/24 h, in contrast to 0.13 mmol/24 h in cases. The latter also presented with a greater decrease in urinary excretion of oxalate and the ion-activity product index of calcium oxalate.

Among all stone formers in both groups, eGFR and the total number of stone events correlated significantly and negatively (R = −0.314, *p* = 0.030). In addition, a significant positive correlation was noted between eGFR and urine pH value (R = 0.429, *p* < 0.001), as well as between eGFR and urinary citrate excretion under the balanced diet (R = 0.308, *p* = 0.008). No significant correlation was detected between eGFR and urinary oxalate excretion under the balanced diet (R = −0.215, *p* = 0.066). Moreover, eGFR correlated significantly and negatively with the relative supersaturation of uric acid (R = −0.303, *p* = 0.009) and with the ion-activity product of uric acid under the balanced diet (R = −0.313, *p* = 0.007). No other significant associations were detected between eGFR and urinary parameters under the balanced, standardized diet.

Under the balanced standardized diet, low urine volume and pH value, hypomagnesuria, hyperoxaluria, and hypocitraturia appeared more often in the case group (Table 4). Hypercalciuria was diagnosed more frequently in the control group, with urinary calcium excretion exceeding 5 mmol/24 h in 45.9% of controls (including one patient with urinary calcium excretion greater than 8 mmol/24 h), in contrast to only two patients (5.4%) with hypercalciuria in the case group. Cases had a 93.7% lower risk of being diagnosed with hypercalciuria than controls. No significant differences between the two groups were found for hyperuricosuria.

### 3.3. Dietary Intake

Table 5 shows the nutrient intake of cases and matched controls on their habitual diets and on the balanced, standardized diet. The changes in nutrient intake resulting from the switch to a balanced diet did not differ significantly between the cases and the controls.

### 3.4. Multivariable Linear Regression Model for Glomerular Filtration Rate

**Figure 1:**
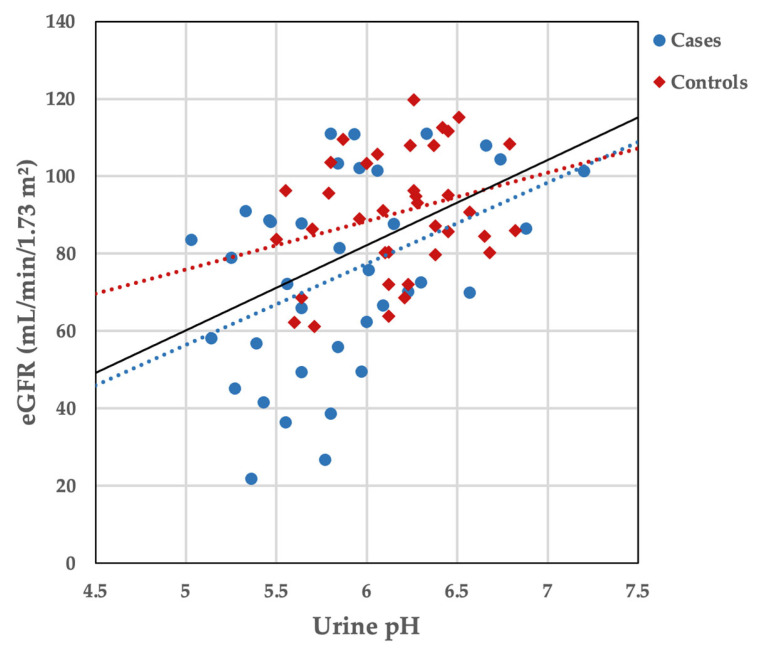
Relationship between urine pH under the balanced, standardized diet and eGFR. The black line represents the expected eGFR conditional on urine pH, estimated from a univariate regression model, while the blue and red dotted lines represent this relationship for the subgroups of cases and controls, respectively. Abbreviation: eGFR, estimated glomerular filtration rate.

Multivariable linear regression analysis was performed for all stone formers in both groups to identify potential factors influencing eGFR (Table 6). The total number of stone events, including both symptomatic and asymptomatic events based on the patient history, as well as urine pH and urinary oxalate excretion under the balanced, standardized diet, were included in the regression model as independent variables of interest. Their effects were additionally adjusted for BMI, patient group, presence of hypertension, and presence of diabetes mellitus. Neither the total number of stone events nor urinary oxalate excretion were found to have a significant association with eGFR. In contrast, a significant relationship between urine pH and eGFR was identified, with a 0.5-unit increase in pH associated with an expected increase of 7.4 mL/min/1.73 m^2^ in eGFR. As a sensitivity analysis, the model was fitted to the unimputed data set and to imputed data sets from which patients with the most extreme urine pH values (lower 5%, upper 5%, lower 2.5%, and upper 2.5%) were removed. The significant positive association between urine pH and eGFR was consistently observed in all settings. Additionally, no significant difference in the effect of urine pH between cases and controls was found (*p* = 0.421). The relationship between urine pH and eGFR is presented as a scatter plot in Figure 1.

## 4. Discussion

Hyperoxaluria is a major risk factor for calcium oxalate stone disease [5]. Even minor alterations in urinary oxalate concentration have a significant impact on calcium oxalate crystallization. Therefore, changes in oxalate concentration affect the urinary solubility product of calcium oxalate more than an equimolar change in calcium concentration [38]. In the present study, urinary oxalate excretion was significantly higher in calcium oxalate stone formers with enteric hyperoxaluria on both diets, with a greater reduction observed on the balanced diet. It is noteworthy that hyperoxaluria was still present in 73.0% of these patients under the balanced, standardized diet, compared with 2.7% of idiopathic calcium oxalate stone formers, even though no difference in dietary oxalate intake was found between the groups. The [^13^C_2_] oxalate absorption test, conducted under controlled, standardized conditions, revealed a significant difference in the median [^13^C_2_] oxalate absorption of 14.8% in stone formers with enteric hyperoxaluria and 8.9% in patients without enteric hyperoxaluria. These results are consistent with previous findings suggesting that enteric hyperoxaluria is primarily related to hyperabsorption of oxalate in the intestine [39]. In patients with enteric hyperoxaluria, malabsorptive bowel conditions are associated with an excess of non-absorbed fatty acids due to loss of bile acids with the stool [10]. The binding of these fatty acids to calcium in the gut leads to a lack of free calcium available for oxalate complexation, resulting in increased absorption of oxalate [9,11]. A retrospective study of 297 patients with enteric hyperoxaluria, conducted by D’Costa et al., estimated that a 20% decrease in urinary oxalate excretion would be associated with 25% lower odds of a subsequent stone event [40]. Since dietary oxalate is expected to contribute to a greater extent to urinary oxalate excretion in patients with elevated intestinal oxalate absorption, patients with enteric hyperoxaluria should strictly limit their oxalate intake. Therefore, foods and beverages with high amounts of oxalate, such as spinach, rhubarb, sorrel, mangold, sesame, cocoa, sweet potatoes, okra, and green, black and iced teas, should be avoided. Foods with moderate oxalate content, such as legumes and nuts, should be restricted [27]. Dietary advice is highly important to provide information on foods high in oxalate content and ensure patients’ adherence.

Both calcium and magnesium can reduce intestinal oxalate absorption by forming complexes with oxalate and therefore diminish urinary oxalate excretion [41]. Urinary calcium excretion of idiopathic calcium oxalate stone formers was found to be twice as high as that observed in patients with enteric hyperoxaluria on the free-choice diet, and remained higher even under the balanced, standardized diet. Since dietary calcium intake did not differ between the two groups under either diet, it is hypothesized that the higher urinary calcium excretion can be attributed to higher intestinal calcium absorption in idiopathic calcium oxalate stone formers. Hyperabsorption of calcium was indeed present in 27.0% of idiopathic stone formers, but only in 2.7% of stone formers with enteric hyperoxaluria. Additionally, patients without enteric hyperoxaluria experienced a far greater decline in urinary calcium excretion of 1.24 mmol/24 h, in contrast to 0.13 mmol/24 h in stone formers with enteric hyperoxaluria. In patients with intestinal malabsorption associated with enteric hyperoxaluria, calcium binds to non-absorbed fatty acids and is therefore unavailable for absorption, leading to lower urinary calcium excretion [11]. It is hypothesized that the lower fat content of the balanced, standardized diet may have diminished calcium binding to fatty acids in the bowel lumen. This could result in enhanced complexation of calcium and oxalate and consequently reduced intestinal oxalate absorption [42]. Future studies should examine vitamin D status, parathyroid hormone, and bone turnover markers as potential factors that could also lead to changes in calcium metabolism. Calcium restriction is not recommended for patients with idiopathic calcium oxalate stone formation, since a normal-calcium, low-protein, low-salt diet has been demonstrated to reduce the risk of stone recurrence more effectively than a low-calcium diet [43]. While idiopathic calcium oxalate stone formers should ensure that their daily calcium intake is within the recommended range of 1000 to 1200 mg [44], calcium supplementation is advised for calcium oxalate stone formers with enteric hyperoxaluria [10,29].

Patients suffering from malabsorptive bowel conditions are prone to developing hypomagnesuria due to impaired intestinal magnesium uptake [45]. Despite similar dietary magnesium intake under both diets, urinary magnesium excretion was significantly lower in calcium oxalate stone formers with enteric hyperoxaluria. Under the balanced, standardized diet, hypomagnesuria was present in 70% of these patients, compared to 14% of patients without enteric hyperoxaluria. Because magnesium binds to oxalate and thereby diminishes oxalate absorption in the intestine and urinary oxalate excretion, magnesium supplementation should be considered as a treatment option for enteric hyperoxaluria [39]. Supplementation with calcium and magnesium has been shown to significantly reduce intestinal oxalate absorption, with calcium supplementation being more than twice as effective as magnesium supplementation [41].

Low urine volume is one of the most critical risk factors for calcium oxalate stone formation [46]. In the present study, the median urine volume was below 2 L under the habitual diet in both groups and did not differ significantly between the groups. Even under the balanced, standardized diet, 41% of stone formers with enteric hyperoxaluria still did not meet the recommended urine volume of at least 2 L. In contrast, only 16% of idiopathic calcium oxalate stone formers were noted to have diminished urine volume. This may be due to fecal fluid loss in patients suffering from enteric hyperoxaluria affected by malabsorptive intestinal conditions [10]. Therefore, it is particularly important for calcium oxalate stone formers with fluid loss due to malabsorption to ensure adequate fluid intake throughout the day and to achieve sufficient urine volume in order to reduce the risk of stone formation and recurrence [27,46].

In both calcium oxalate stone formers with and without enteric hyperoxaluria, dietary intervention resulted in a decrease in the relative supersaturation of calcium oxalate and the ion-activity product index of calcium oxalate. The decline in the ion-activity product index of calcium oxalate was found to be significantly greater in patients with enteric hyperoxaluria, which underlines that these patients could particularly benefit from maintaining a balanced diet. Additionally, the ion-activity product of uric acid was significantly higher in stone formers with enteric hyperoxaluria under both diets, which may be attributed to lower urine pH in this cohort.

Kidney stone patients are at increased risk of developing CKD and end-stage kidney disease [17], with patients suffering from malabsorptive bowel diseases being at particular risk of impaired kidney function [18]. A study conducted by Puurunen et al. noted that a greater proportion of patients with malabsorptive conditions had prevalent CKD than those in the overall population [47]. This is in alignment with the findings of the present study, in which eGFR was significantly lower in calcium oxalate stone formers with enteric hyperoxaluria than in idiopathic calcium oxalate stone formers. Therefore, identifying risk factors and indicators for CKD and developing strategies to prevent the appearance and progression of CKD associated with stones is of the utmost importance, especially for patients with malabsorptive bowel conditions. Reduced kidney function may be attributed to the consequences of stone events, such as obstruction, the underlying disease causing urolithiasis, and therapeutic interventions for stone removal [16,17]. In this matched case-control study, a multivariable linear regression model was used to evaluate factors associated with impaired kidney function in stone formers. Interestingly, multivariable linear regression analysis revealed no significant association between eGFR and the total number of stone events prior to the study, which was initially assumed to be a marker of stone disease severity. Puurunen et al. also found that the risk of incident CKD increases with higher urinary oxalate excretion [47]. Waikar et al. showed that higher urinary oxalate excretion is independently associated with a greater risk of CKD progression and end-stage kidney disease [48]. However, this relationship could not be confirmed in the present study under standardized conditions. No significant association between urinary oxalate excretion under the balanced, standardized diet and eGFR was detected in the multivariable linear regression analysis. However, it should be noted that the statistical power of the regression analysis was limited.

Under standardized conditions, low urine pH occurred more frequently in patients with enteric hyperoxaluria, with a pH of less than 5.8 affecting 43% of stone formers with and 19% of stone formers without enteric hyperoxaluria. Furthermore, the median urine pH was significantly lower under both diets. This could be ascribed to fecal bicarbonate loss due to intestinal malabsorption, resulting in a higher acid load in this cohort [49]. This is further emphasized by the lower blood pH in stone patients with enteric hyperoxaluria, with a median pH of 7.40 in contrast to 7.42 in idiopathic calcium oxalate stone formers, as well as by lower plasma bicarbonate concentration in patients with enteric hyperoxaluria. Diminished luminal and intracellular pH values have been shown to contribute to low urinary citrate excretion by modifying intracellular citrate metabolism and reabsorption [50,51]. Hypocitraturia is recognized as a significant risk factor for calcium oxalate stone disease, since citrate inhibits crystallization through the formation of soluble complexes with calcium [51]. Therefore, the management of hypocitraturia is an important component in the prevention of recurrence of calcium oxalate urolithiasis [52]. This is of even greater relevance in patients with enteric hyperoxaluria presenting with lower urinary citrate excretion than that observed in idiopathic calcium oxalate stone formers under both diets, which is related to metabolic acidosis due to fecal loss of alkali equivalents [10,49,53].

A low urine pH, corresponding to a high urinary proton concentration, is considered to be an independent predictor of CKD [54]. The findings from the present study, utilizing a multivariable linear regression model, revealed a significant association between low urine pH and impaired renal function. Accordingly, a half-unit decrease in urine pH value was associated with an expected loss of 7.4 mL/min/1.73 m^2^ in eGFR. The association between low urine pH and diminished kidney function exists in both directions: On the one hand, it is a well-known mechanism that decreased kidney function leads to a higher acid load and metabolic acidosis, with urinary proton excretion being maintained until very low eGFR levels, resulting in a low urine pH [55,56]. On the other hand, metabolic acidosis has been proposed to promote CKD by diverse mechanisms [21,57]. Consequently, the enhancement of urine pH should be further evaluated as a treatment option for reducing the risk of CKD in calcium oxalate stone formers. However, there is conflicting evidence and recommendations regarding oral alkalization therapy in patients with CKD [20]. The KDIGO 2024 Clinical Practice Guideline for the Evaluation and Management of Chronic Kidney Disease recommends pharmacological treatment of metabolic acidosis only for patients with serum bicarbonate levels below 18 mmol/L, as several studies have found no significant effect of bicarbonate supplementation on kidney outcomes [58]. However, the administration of alkaline citrate is recommended for enteric hyperoxaluria, with the aim of raising urine pH and increasing citrate excretion [44]. In addition, the ingestion of fruit and vegetables constitutes a dietary measure that not only increases urinary citrate excretion without affecting oxalate excretion but may also decrease the relative supersaturation of calcium oxalate [29,59]. Furthermore, a meta-analysis of approximately 122,000 participants who adopted a plant-based diet over an average follow-up time of 11.2 years revealed that a healthy plant-based diet is associated with a 26% lower incidence of CKD [60]. Consequently, particular attention should be paid to low urine pH, as it is hypothesized to serve as a potential indicator of reduced kidney function.

This study has potential limitations. Due to the study design, no causal relationship between urine pH and the risk of CKD could be established. Furthermore, the lack of longitudinal follow-up limits the ability to draw conclusions about the progression of CKD and long-term clinical outcomes. Given the limited number of confounding variables included, the multivariable linear regression model may not be able to fully control for residual confounding. Other variables that were not integrated into the regression model could also be associated with impaired kidney function, such as treatment history and use of medication. This could also affect generalizability, especially given the relatively small sample size. Despite these limitations, this study addresses an important and clinically relevant topic: the metabolic and urinary risk profiles of calcium oxalate stone formers with enteric hyperoxaluria compared with idiopathic calcium oxalate stone formers, evaluated under their habitual diet and a balanced, standardized diet. The standardized dietary intervention is a notable strength, as it reduces dietary variability and allows a clearer interpretation of physiologic differences related to intestinal absorption, acid-base status, and malabsorption. The key findings—persistently higher rates of low urine volume, suboptimal urine pH, hyperoxaluria, hypomagnesuria, and hypocitraturia in calcium oxalate stone formers with enteric hyperoxaluria, even under standardized conditions, and a higher frequency of hypercalciuria among idiopathic calcium oxalate stone formers—are clinically meaningful and align with known pathophysiology.

In addition to a balanced mixed diet, which should form the basis of treatment for enteric hyperoxaluria, certain dietary recommendations may help mitigate the urinary risk profile and reduce the risk of stone formation. The primary objective of dietary management in patients with enteric hyperoxaluria should be the monitoring of urine volume. Generous fluid intake, preferably water, to achieve a urine volume of at least 2.0 to 2.5 L/24 h should be distributed evenly throughout the day and before going to bed [27,44,61,62,63]. Extrarenal fluid losses caused by diarrhea, hot and/or dry environments, occupation, mental stress, and physical activity should be replaced. In addition to the restriction of dietary oxalate, the provision of oral calcium and magnesium supplementation should be considered as a further treatment option for patients suffering from enteric hyperoxaluria [44]. In addition, alkaline citrate can reduce stone formation in enteric hyperoxaluria and is therefore recommended [44]. Further research on calcium oxalate stone formers with enteric hyperoxaluria is needed to evaluate kidney function using additional filtration markers, such as cystatin C, and to assess whether alkalization therapy may improve renal outcomes.

## 5. Conclusions

Elevated supersaturation of calcium oxalate was found to be associated with an increased risk of kidney stone formation and recurrence, which in turn may be linked to an augmented risk of CKD. Therefore, mitigating the urinary risk profile is essential to avoid stone formation and recurrences in calcium oxalate stone formers. As this study shows, dietary treatment of urolithiasis is an important component in the management of stone disease. Patients should be trained in the importance of maintaining a sufficient urine volume of at least 2 L daily and reducing dietary oxalate intake as part of a balanced diet. It is clinically relevant that patients with enteric hyperoxaluria, even while following a balanced diet, more frequently continued to present with hyperoxaluria, hypocitraturia, hypomagnesuria, low urine volume, and low urine pH. These findings indicate the need for targeted dietary and metabolic management, including a potential role for calcium, magnesium, and citrate supplementation in this patient group. As low urine pH is hypothesized to potentially indicate reduced kidney function, particular attention should be paid to this parameter at initial diagnosis and during follow-up. Further prospective studies are necessary to evaluate the potential and limitations of alkalization therapy for the prevention and treatment of CKD in calcium oxalate stone formers with enteric hyperoxaluria.

## Figures and Tables

**Table 1:** Anthropometric, clinical, and metabolic characteristics of calcium oxalate stone formers with enteric hyperoxaluria and idiopathic calcium oxalate stone formers.

	Cases Median (IQR) *n* (%)	Controls Median (IQR) *n* (%)	*p* Value
Number of patients	37	37	
Gender (women/men)	10/27 (27.0%/73.0%)	10/27 (27.0%/73.0%)	1.000
Age (years)	48 (20)	46 (19)	0.442
Height (cm)	178 (8) ^a^	174 (7)	0.009
Weight (kg)	75.3 (24.3) ^a^	80.0 (14.5)	0.208
BMI (kg/m^2^)	24.6 (6.9) ^a^	26.6 (4.6)	0.016
BMI < 18.5 kg/m^2^	1/36 (2.8%)	0/37 (0.0%)	
BMI 18.5–24.9 kg/m^2^	19/36 (52.8%)	13/37 (35.1%)	
BMI 25.0–29.9 kg/m^2^	13/36 (36.1%)	20/37 (54.1%)	
BMI ≥ 30.0 kg/m^2^	3/36 (8.3%)	4/37 (10.8%)	
Cardiovascular diseases			
Hypertension	8/37 (21.6%)	9/37 (24.3%)	1.000
Myocardial infarction	1/37 (2.7%)	1/37 (2.7%)	1.000
Stroke/TIA	1/37 (2.7%)	1/37 (2.7%)	1.000
Type 2 diabetes	2/37 (5.4%)	3/37 (8.1%)	1.000
Gallstone/cholecystectomy	11/37 (29.7%)	4/37 (10.8%)	0.065
Oxalate absorption (%)	14.8 (13.0) ^b^	8.9 (7.1) ^c^	0.002
Oxalate absorption ≥ 10.0%	24/31 (77.4%)	13/29 (44.8%)	0.035
Hypercalciuria	2/37 (5.4%)	17/37 (45.9%)	<0.001
Absorptive hypercalciuria ^d^	1/37 (2.7%)	10/37 (27.0%)	
Renal hypercalciuria ^d^	0/37 (0.0%)	3/37 (8.1%)	
Idiopathic hypercalciuria ^d^	1/37 (2.7%)	4/37 (10.8%)	
eGFR (mL/min/1.73 m^2^)	75.7 (39.9)	90.7 (24.4)	0.004
eGFR ≥ 90.0 mL/min/1.73 m^2^	10/37 (27.0%)	19/37 (51.4%)	
eGFR 60.0–89.9 mL/min/1.73 m^2^	16/37 (43.2%)	18/37 (48.6%)	
eGFR 45.0–59.9 mL/min/1.73 m^2^	6/37 (16.2%)	0/37 (0.0%)	
eGFR 30.0–44.9 mL/min/1.73 m^2^	3/37 (8.1%)	0/37 (0.0%)	
eGFR 15.0–29.9 mL/min/1.73 m^2^	2/37 (5.4%)	0/37 (0.0%)	
Blood gas analysis			
pH	7.40 (0.04) ^e^	7.42 (0.02) ^f^	0.001
Bicarbonate (mmol/L)	23.2 (2.5) ^e^	25.2 (1.3) ^f^	0.012
Base excess	−1.6 (2.8) ^e^	0.8 (1.9) ^f^	0.001
Metabolic acidosis ^g^	4/24 (16.7%)	0/26 (0.0%)	0.135

Abbreviations: BMI, body mass index; eGFR, estimated glomerular filtration rate; IQR, interquartile range; TIA, transient ischemic attack. ^a^ *n* = 36. ^b^ *n* = 31. ^c^ *n* = 29. ^d^ determined according to the calcium loading test. ^e^ *n* = 24. ^f^ *n* = 26. ^g^ defined as bicarbonate < 22 mmol/L.

**Table 2:** Medical history of urinary stone disease of calcium oxalate stone formers with enteric hyperoxaluria and idiopathic calcium oxalate stone formers.

	Cases Median (IQR) *n* (%)	Controls Median (IQR) *n* (%)	*p* Value
Age at first stone event (years)	39 (18)	32 (18)	0.201
Duration of stone disease (years)	9 (14)	12 (18)	0.213
Number of stone events in the past year	4 (28) ^a^	2 (4) ^b^	0.067
Number of stone events in total	20 (45) ^c^	12 (29) ^d^	0.444
Anatomic anomalies	10/37 (27.0%)	11/37 (29.7%)	1.000
Stenosis	3/37 (8.1%)	1/37 (2.7%)	
Kidney cysts	5/37 (13.5%)	6/37 (16.2%)	
Partial nephrectomy	0/37 (0.0%)	1/37 (2.7%)	
Total nephrectomy	1/37 (2.7%)	0/37 (0.0%)	
Total nephrectomy and kidney cysts	1/37 (2.7%)	0/37 (0.0%)	
Total nephrectomy and stenosis	0/37 (0.0%)	1/37 (2.7%)	
Pelvic kidney and kidney cysts	0/37 (0.0%)	1/37 (2.7%)	
Duplex kidney and kidney cysts	0/37 (0.0%)	1/37 (2.7%)	
Laterality			0.804
Bilateral	26/37 (70.3%)	28/37 (75.7%)	
Unilateral	11/37 (29.7%)	9/37 (24.3%)	
Type of stone removal			
Spontaneous passage	28/37 (75.7%)	36/37 (97.3%)	0.021
Spontaneous passage alone	4/37 (10.8%)	4/37 (10.8%)	1.000
ESWL	25/37 (67.6%)	29/37 (78.4%)	0.454
Ureteroscopy	21/37 (56.8%)	20/37 (54.1%)	1.000
Percutaneous nephrolithotomy	10/37 (27.0%)	3/37 (8.1%)	0.065
Open surgery	4/37 (10.8%)	5/37 (13.5%)	1.0000

Abbreviations: ESWL, extracorporeal shock wave lithotripsy; IQR, interquartile range. ^a^ *n* = 27. ^b^ *n* = 29. ^c^ *n* = 26. ^d^ *n* = 22.

**Table 3:** Urinary parameters of calcium oxalate stone formers with enteric hyperoxaluria and idiopathic calcium oxalate stone formers under the habitual diet and the balanced diet.

	Cases *n* = 37 Median (IQR)			Controls *n* = 37 Median (IQR)			Cases vs. Controls *p* Value		
	Habitual Diet	Balanced Diet	Difference	Habitual Diet	Balanced Diet	Difference	Habitual Diet	Balanced Diet	Difference
Volume	1.680 (1.020)	2.060 (0.878)	0.420 (1.503)	1.900 (1.090)	2.370 (0.575)	0.550 (1.110)	0.175	0.180	0.563
pH	5.68 (0.46)	5.84 (0.66)	0.16 (0.61)	6.14 (0.75)	6.23 (0.52)	0.08 (0.58)	<0.001	0.002	0.599
Density (g/cm^3^)	1.010 (0.006)	1.006 (0.004)	−0.004 (0.005)	1.010 (0.007)	1.005 (0.002)	−0.005 (0.008)	0.777	0.343	0.777
Sodium (mmol/24 h)	150 (113)	113 (62)	−36 (119)	159 (91)	104 (40)	−51 (69)	0.870	0.114	0.464
Potassium (mmol/24 h)	45 (22)	45 (30)	5 (20)	51 (27)	58 (21)	6 (26)	0.024	0.063	0.709
Calcium (mmol/24 h)	2.68 (2.75)	2.63 (1.92)	−0.13 (1.25)	5.42 (5.05)	4.22 (3.48)	−1.24 (2.55)	<0.001	<0.001	0.002
Magnesium (mmol/24 h)	1.81 (1.31)	2.28 (1.67)	0.54 (0.81)	4.11 (2.42)	4.48 (2.01)	0.12 (1.27)	<0.001	<0.001	0.158
Ammonium (mmol/24 h)	42.2 (27.3) ^a^	32.3 (18.8) ^a^	−3.3 (26.6) ^a^	25.7 (18.6)	24.8 (13.4)	−5.2 (13.5)	0.017	0.002	0.471
Chloride (mmol/24 h)	181 (130)	129 (70)	−55 (132)	153 (88)	102 (43)	−59 (63)	0.182	0.031	0.893
Phosphate (mmol/24 h)	27.1 (10.8)	24.8 (7.5)	−1.9 (9.1)	27.7 (13.1)	25.6 (8.6)	−1.0 (12.7)	0.612	0.492	0.743
Sulfate (mmol/24 h)	16.9 (9.1)	14.5 (5.9)	−1.7 (8.2)	20.2 (7.8)	16.8 (4.3)	−2.5 (6.5)	<0.001	<0.001	0.162
Creatinine (mmol/24 h)	13.52 (4.85)	13.41 (5.05)	−0.41 (2.75)	13.78 (5.61)	13.34 (5.67)	−0.73 (2.52)	0.202	0.172	0.882
Uric acid (mmol/24 h)	2.79 (1.19)	2.49 (1.07)	−0.35 (1.10)	3.80 (1.43)	3.18 (0.70)	−0.36 (1.09)	<0.001	<0.001	0.323
Oxalate (mmol/24 h)	0.702 (0.575)	0.574 (0.365)	−0.179 (0.279)	0.350 (0.135)	0.284 (0.082)	−0.057 (0.160)	<0.001	<0.001	0.035
Citrate (mmol/24 h)	0.343 (0.964)	0.857 (2.301)	0.212 (0.887)	2.355 (1.955)	2.834 (2.028)	0.211 (1.163)	<0.001	<0.001	0.893
RS Uric acid	1.700 (1.974)	1.034 (1.179)	−0.593 (1.679)	1.135 (2.097)	0.610 (0.972)	−0.459 (1.715)	0.060	0.019	0.437
RS CaOx	9.574 (5.397)	5.281 (3.730)	−2.869 (5.289)	5.802 (3.564)	3.384 (2.168)	−1.804 (3.910)	0.001	0.001	0.206
AP Uric acid (10^−9^)	1.165 (1.457)	0.667 (0.854)	−0.377 (1.178)	0.734 (1.457)	0.380 (0.634)	−0.296 (1.200)	0.047	0.015	0.370
AP CaOx index	2.201 (1.478)	1.040 (0.961)	−0.961 (1.477)	1.020 (0.734)	0.557 (0.399)	−0.468 (0.742)	<0.001	<0.001	0.009

Abbreviations: AP, activity product; IQR, interquartile range; RS, relative supersaturation. ^a^ *n* = 35.

**Table 4:** Urinary abnormalities of calcium oxalate stone formers with enteric hyperoxaluria and idiopathic calcium oxalate stone formers under the balanced, standardized diet.

	Reference Range	Cases *n* = 37 *n* (%)	Controls *n* = 37 *n* (%)	Odds Ratio [95% CI]	*p* Value
Volume	<2.000 L/24 h	15 (40.5%)	6 (16.2%)	0.100 [0.013, 0.781]	0.012
	≥2.000 L/24 h	22 (59.5%)	31 (83.8%)		
pH	<5.80	16 (43.2%)	7 (18.9%)	0.250 [0.071, 0.886]	0.035
	≥5.80	21 (56.8%)	30 (81.1%)		
Calcium	<5.00 mmol/24 h	35 (94.6%)	20 (54.1%)	0.063 [0.008, 0.471]	<0.001
	≥5.00 mmol/24 h	2 (5.4%)	17 (45.9%)		
Magnesium	<3.00 mmol/24 h	26 (70.3%)	5 (13.5%)	0.045 [0.006, 0.337]	<0.001
	≥3.00 mmol/24 h	11 (29.7%)	32 (86.5%)		
Uric acid	<4.00 mmol/24 h	34 (91.9%)	31 (83.8%)	0.400 [0.078, 2.062]	0.453
	≥4.00 mmol/24 h	3 (8.1%)	6 (16.2%)		
Oxalate	<0.45 mmol/24 h	10 (27.0%)	36 (97.3%)	25.000 [3.388, 184.501]	<0.001
	≥0.45 mmol/24 h	27 (73.0%)	1 (2.7%)		
Citrate	<1.700 mmol/24 h	24 (64.9%)	6 (16.2%)	0.143 [0.043, 0.479]	<0.001
	≥1.700 mmol/24 h	13 (35.1%)	31 (83.8%)		

Abbreviation: CI, confidence interval. The odds ratios refer to the odds for the higher value categories, where an odds ratio greater than 1 indicates that the odds of falling into the higher value category are increased in cases compared to controls.

**Table 5:** Nutrient intake of calcium oxalate stone formers with enteric hyperoxaluria and idiopathic calcium oxalate stone formers under the habitual diet and under the balanced diet.

	Cases *n* = 31 Median (IQR)			Controls *n* = 32 Median (IQR)			Cases vs. Controls *p* Value
	Habitual Diet	Balanced Diet	Pre-Post Difference ^a^	Habitual Diet	Balanced Diet	Pre-Post Difference ^a^	Pre-Post Difference ^b^
Energy (kcal/day)	2548 (1001)	2355	−193 (1001) *	2211 (790)	2355	144 (790)	0.129
Protein (g/day)	97 (42)	71	−26 (42) *	83 (31)	71	−12 (31) *	0.423
Fat (g/day)	92 (61)	81	−11 (61) *	80 (31)	81	1 (31)	0.062
Carbohydrates (g/day)	293 (82)	327	34 (82)	257 (111)	327	70 (111) *	0.353
Fiber (g/day)	22 (10)	31	9 (10) *	21 (12)	31	10 (12) *	0.671
Sodium (mg/day)	3452 (2519)	2300	−1152 (2519) *	3457 (2118)	2300	−1157 (2118) *	0.532
Potassium (mg/day)	3275 (1795)	3390	115 (1795)	3005 (1428)	3390	385 (1428) *	0.515
Calcium (mg/day)	890 (345)	977	87 (345)	883 (369)	977	94 (369)	0.940
Magnesium (mg/day)	417 (208)	341	−76 (208) *	376 (131)	341	−35 (131) *	0.901
Phosphorus (mg/day)	1427 (656)	1432	6 (656)	1337 (504)	1432	95 (504)	0.565
Methionine (mg/day)	2027 (771)	1415	−612 (771) *	1714 (761)	1415	−299 (761) *	0.227
Cysteine (mg/day)	1270 (597)	835	−435 (597) *	1089 (431)	835	−254 (431) *	0.190
Total oxalate (mg/day)	151 (118)	121	−30 (118) *	150 (172)	121	−29 (172) *	0.423
Soluble oxalate (mg/day)	68 (40)	54	−14 (40) *	70 (38)	54	−16 (38) *	0.408
Purines (mg/day)	449 (340)	449	0 (340)	407 (277)	449	42 (277)	0.315
Cholesterol (mg/day)	376 (294)	195	−181 (294) *	353 (128)	195	−158 (128) *	0.380
MUFA (g/day)	34 (24)	26	−8 (24) *	30 (16)	26	−4 (16)	0.094
PUFA (g/day)	12 (7)	19	7 (7) *	12 (5)	19	7 (5) *	0.291
Water (mL/day)	3130 (1268)	3437	307 (1268)	3038 (1252)	3437	399 (1252)	0.353
Alcohol (g/day)	5 (20)	0	−5 (20) *	2 (17)	0	−2 (17) *	0.392

Abbreviations: IQR, interquartile range; MUFA, monounsaturated fatty acids; PUFA, polyunsaturated fatty acids. Wilcoxon signed rank test within groups, habitual diet vs. balanced diet: * *p* < 0.05. ^a^ calculated as balanced diet minus habitual diet. ^b^ test for a difference in the pre-post differences (differences-in-differences) in nutrient intake between cases and matched controls, using Wilcoxon signed rank test.

**Table 6:** Multivariable linear regression model for estimated glomerular filtration rate.

Variable	Coefficient	95% CI	*p* Value
(Intercept)	5.165	[−76.749, 87.079]	0.900
Total number of stone events	−0.079	[−1.026, 0.869]	0.868
Urine pH	14.862	[3.735, 25.989]	0.010
Urinary oxalate excretion (mmol/24 h)	8.907	[−12.249, 30.063]	0.403
BMI (kg/m^2^)	−0.216	[−1.452, 1.020]	0.728
Patient group (cases)	−15.001	[−28.290, −1.713]	0.028
Hypertension (presence)	−15.815	[−28.309, −3.321]	0.014
Diabetes mellitus (presence)	15.698	[−3.492, 34.888]	0.107

Abbreviations: BMI, body mass index; CI, confidence interval. R^2^ = 0.356. *p* Value < 0.001. Pooled results based on 50 imputed data sets with *n* = 74.

## Data Availability

The data presented in the study are available upon reasonable personal request due to data privacy reasons.

## Abbreviations

The following abbreviations are used in this manuscript:

AP	Activity product
BMI	Body mass index
CI	Confidence interval
CKD	Chronic kidney disease
eGFR	Estimated glomerular filtration rate
ESWL	Extracorporeal shock wave lithotripsy
IQR	Interquartile range
MUFA	Monounsaturated fatty acids
PUFA	Polyunsaturated fatty acids
RS	Relative supersaturation
TIA	Transient ischemic attack
